# RF_Purify: a novel tool for comprehensive analysis of tumor-purity in methylation array data based on random forest regression

**DOI:** 10.1186/s12859-019-3014-z

**Published:** 2019-08-16

**Authors:** Pascal David Johann, Natalie Jäger, Stefan M. Pfister, Martin Sill

**Affiliations:** 1 Division of Pediatric Neurooncology, German Cancer Research Center (DKFZ), Heidelberg, Germany; 2 Hopp Children’s Cancer Center (KiTZ), Heidelberg, Germany; 3 Department of Pediatric Hematology and Oncology, University Children’s Hospital Heidelberg, Heidelberg, Germany; 4 German Cancer Consortium (DKTK), German Cancer Research Center (DKFZ), Heidelberg, Germany

## Abstract

**Background:**

With the advent of array-based techniques to measure methylation levels in primary tumor samples, systematic investigations of methylomes have widely been performed on a large number of tumor entities. Most of these approaches are not based on measuring individual cell methylation but rather the bulk tumor sample DNA, which contains a mixture of tumor cells, infiltrating immune cells and other stromal components. This raises questions about the purity of a certain tumor sample, given the varying degrees of stromal infiltration in different entities. Previous methods to infer tumor purity require or are based on the use of matching control samples which are rarely available. Here we present a novel, reference free method to quantify tumor purity, based on two Random Forest classifiers, which were trained on ABSOLUTE as well as ESTIMATE purity values from TCGA tumor samples. We subsequently apply this method to a previously published, large dataset of brain tumors, proving that these models perform well in datasets that have not been characterized with respect to tumor purity .

**Results:**

Using two gold standard methods to infer purity – the ABSOLUTE score based on whole genome sequencing data and the ESTIMATE score based on gene expression data- we have optimized Random Forest classifiers to predict tumor purity in entities that were contained in the TCGA project. We validated these classifiers using an independent test data set and cross-compared it to other methods which have been applied to the TCGA datasets (such as ESTIMATE and LUMP).

Using Illumina methylation array data of brain tumor entities (as published in Capper et al. (Nature 555:469-474,2018)) we applied this model to estimate tumor purity and find that subgroups of brain tumors display substantial differences in tumor purity.

**Conclusions:**

Random forest- based tumor purity prediction is a well suited tool to extrapolate gold standard measures of purity to novel methylation array datasets. In contrast to other available methylation based tumor purity estimation methods, our classifiers do not need a priori knowledge about the tumor entity or matching control tissue to predict tumor purity.

**Electronic supplementary material:**

The online version of this article (10.1186/s12859-019-3014-z) contains supplementary material, which is available to authorized users.

## Background

Tumors represent a complex milieu that does not only comprise the malignant cells themselves but receives contributions from different cell types: Invading immune cells as a part of the hosts’ defense against the tumor, blood vessels, fibroblasts and other non-neoplastic cells constitute the tumor microenvironment. The usual procedure to investigate tumor DNA is the isolation from samples after surgical removal. Thus, the DNA which is further analyzed contains contaminating cell populations to a varying degree.

Methylation arrays such as the widely used Infinium HumanMethylation450k / MethylationEPIC array have become increasingly popular to classify tumors into clinically meaningful groups based on distinct methylation patterns [1–3]. The array assesses the methylation levels of mainly promoter based cytosin residues in the genome.

These unsupervised and supervised classification procedures are prone to biases coming from methylation patterns other than the ones from tumor cells, such as stromal or immune cells. Thus, it is important to filter out samples with extremely low tumor purities. In addition, when calling DMRs between samples of high versus low tumor purity, the results will be dominated by differences in tumor purity and not genuine DMRs.

In recent years, a number of methods have been published to account for this problem: The ABSOLUTE method has been developed for whole exome sequencing data and is based on measurement of allele frequencies [4]. Unlike other subsequently published techniques which back on the use of normal samples as a reference, the method objectively measures the frequency of somatic aberrations in a specific cancer sample and relates the frequency of these to the whole DNA quantity.

The model has been developed on SNP data from a TCGA dataset which comprises 12 tumor types which have been characterized by different “omics” techniques, including also 450 K methylation arrays. **(**see Additional file 7: Table S3 for entity abbreviations in the TCGA dataset and the dataset derived from Capper et al.)

Although the ABSOLUTE method has been accepted as a standard for SNP data and whole genome sequencing data, its use is currently limited to samples for which either of the datasets is available. A second method, frequently used for gene expression array data, is ESTIMATE which calculates a stromal score and an immune score and combines both to infer tumor purity.

In the following, we present two Random Forest based models which permit to extrapolate both ESTIMATE and ABSOLUTE purity estimates on novel tumor methylation array datasets. Our approach differs from more recently published methods InfiniumPurify and PAMES in that it accepts the two methods (ESTIMATE and ABSOLUTE) as the gold standard for purity estimation while InfiniumPurify is based on identifying differentially methylated regions between tumor and normal samples which can be used to infer purity via a kernel density estimation. Although a control-free variant of the approach has been published recently [5], this is only applicable for entities which are represented in the TCGA datasets and can not be applied to e.g. entities from the pediatric spectrum which we have examined here and where no non-neoplastic tissue samples are available [5]. Thus this method cannot be applied to study the purity in our dataset derived from Capper et al. 2018 [1].

PAMES (Purity Assessment from clonal Methylation Sites) builds on a number of conserved CG sites identified in the TCGA dataset to infer tumor purity [6]. One concern about this method is that it may overrate tumor purity estimation as only few samples from the TCGA dataset reached tumor purity estimates below 0.9 which is in contrast to previous assessments of tumor purity, indicating a much wider range of tumor purities in this dataset [7]. (Table 1).

**Table 1:** Overview on published methods to infer tumor purity based on WES/SNP array, gene expression arrays and methylation arrays

Publication	Method name	Statistical framework/technique	Datasets used for establishing the method/ validation of the method	Datatypes which can be used as input
Carter et al. [4]	ABSOLUTE	Tumor purity inference based on somatic copy number aberrations in SNP arrays	TCGA	WES data/SNP array
Yoshihara et al., 2013 [8]	ESTIMATE	Comparison of various published gene sets to delineate a) immune signature b) stromal signature - based on these signatures, calculation of purity score	TCGA	Affymetrix gene expression array data
Aran, D. et al. 2015 [7]	LUMP (leukocytes unmethylation for purity)	Averaging of the methylation values 44 CpG sites, known to be hypomethylated in immune cells	TCGA	450 K methylation array data
Zhang et al. 2017 [9], Qin al. 2018 [5]	InfiniumPurify	Tumor purity estimation: (PMID:28122605) comparison of tumor and normal samples to identify DMC (differentially methylated CpG sites) between tumors and an universal set of normal samples in the TCGA dataset followed by kernel density estimation to obtain tumor purity	TCGA	450 K Methylation array data
Benelli et al. 2018 [6]	PAMES (Purity Assessment from clonal MEthylation Sites)	- Calculation of average methylation values per CpG island from TCGA entities. - Calculation of the Area under the curve for the ROC curves of each CpG island: If AUC < 0.2 or AUC > 0.8 a certain CpG site was considered discriminatory and taken into the model. - Tumor purity estimate based on the median of hypomethylated and hypermethylated sites	TCGA (generation of the model), Comparison to other TCGA samples and one additional dataset (333 prostata adenocarcinomas)	450 K methylation array data

As a general setback of all these models, to the best of our knowledge only the PAMES method has been validated outside the TCGA dataset and none of these methods has been applied in rare entities that are not represented in TCGA.

In addition, no emphasis has been laid so far on the comparison of different tumor subgroups: It has been known for several years that e.g. breast cancer and glioblastomas consist [10] of different tumor subgroups with distinct clinical features and probably also different cells of origin. For the latter, it has even been shown that the neural subtype may be defined solely by stromal or non-neoplastic tissue contamination [11].

The cell of origin is particularly important when non-neoplastic controls are chosen in whole genome characterization experiments, as these samples do not represent a proper physiological control but are themselves a mixture of different non-neoplastic cell types. Our group and others have generated an extensive dataset of tumors – enriched for pediatric brain tumors- which have so far not been systematically investigated with respect to their purity. Aiming to estimate ABSOLUTE and ESTIMATE tumor purity in methlyation array data sets beyond the TCGA data set, we trained Random Forest regression models, that automatically perform selection of CpG sites important for the prediction and do not rely on supervised differentially methylation analysis between tumor versus normal tissue.

Therefore, two Random Forest models were trained, the first on the ABSOLUTE and the second on the ESTIMATE values derived from TCGA data and subsequently applied to the dataset in Capper et al. (2018, 1]. Both 450 K methylation data and ABSOLUTE values are available for in total 2310 of the TCGA samples and served as a training and test cohort for the Random Forest model. For the ESTIMATE based model, the training and test set comprised 6360 samples. We cross-compared both our ABSOLUTE and our ESTIMATE based RF models to other purity measures which were available in the TCGA dataset (such as LUMP).

Subsequently, we applied the model to the dataset published in Capper et al. (2018) to delineate tumor purities in this large set of pediatric brain tumors.

## Results

### Validation of the random Forest classifier to predict tumor purity in the TCGA data

**Fig. 1:**
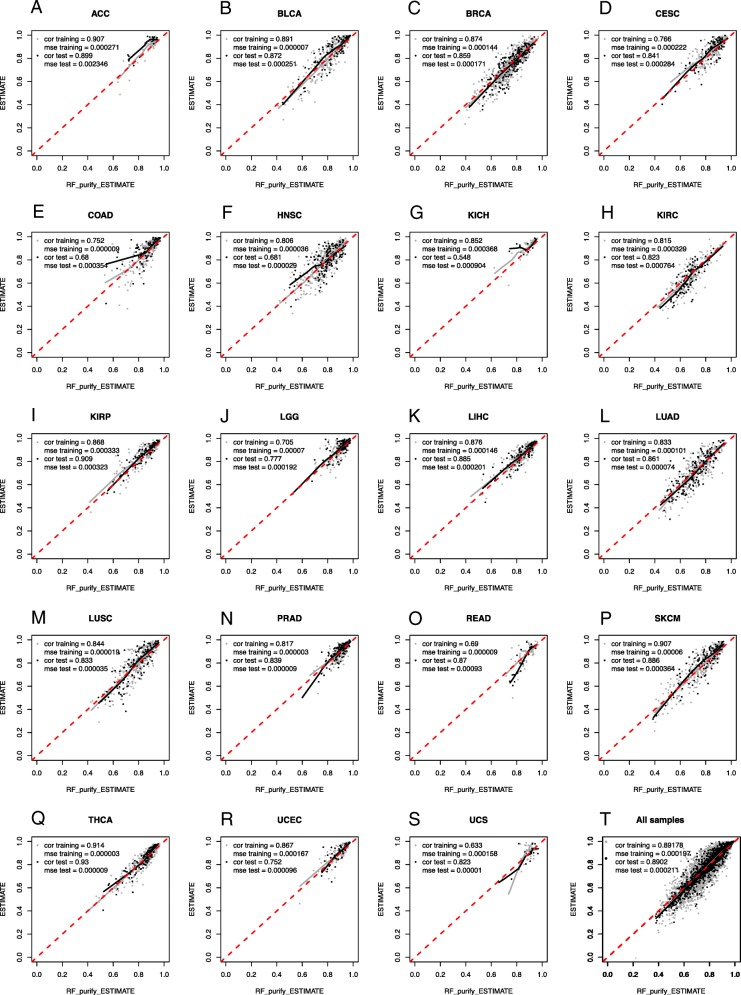
Pearson correlation of the ESTIMATE purity values and RF_Purify_ESTIMATE for the different TCGA tumor entities, split into training and test set (**a**-**s**) and for the whole TCGA set with ESTIMATE values available (**t**)

After having established two RF models as described in the methods section, we empirically compared the correlation and mean squared error of RF_Purify_ABSOLUTE/RF_Purify_ESTIMATE (Fig. 1and Additional file 1: Figure S1) with the ABSOLUTE and ESTIMATE values of different entities represented in the TCGA dataset respectively.

Overall, we found a tight correlation between either the published ABSOLUTE or ESTIMATE values and the RF predicted tumor purities. Moreover, there was no systematic bias for the new model to either over−/ or underestimate tumor purity in a given entity and no overfitting of the training data.

Figure 1 displays the correlation of RF_Purify_ESTIMATE and Additional file 1: Figure S1 the correlations of RF_Purify_ABSOLUTE with the TCGA dataset split by entity.

The global differences in purity between the different tumor types are preserved when comparing the two methods – the average tumor purity of the ESTIMATE method is higher than for the ABSOLUTE method.

**Fig. 2:**
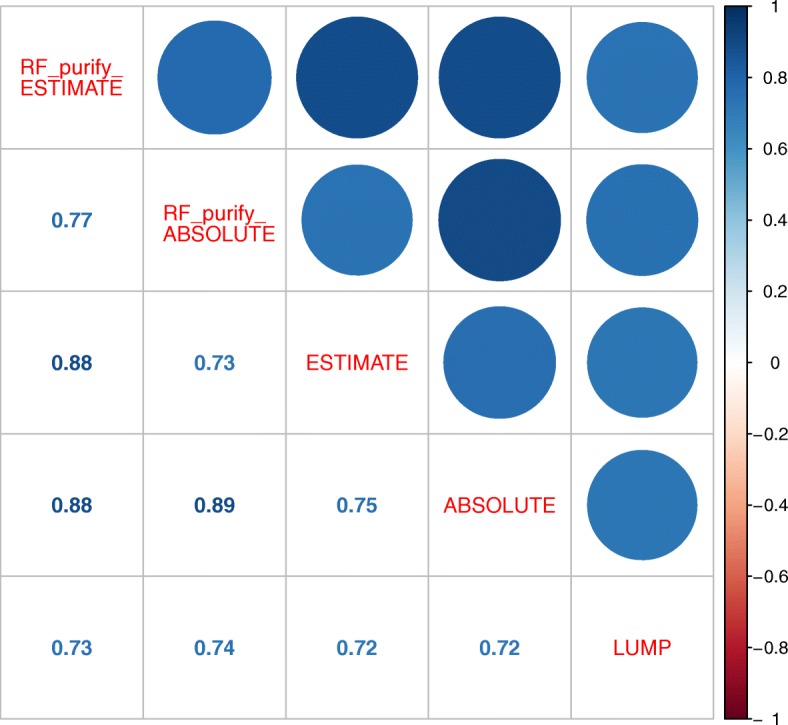
Dot plot visualizing the pearson correlation of tumor purities assessed by RF_Purify_ESTIMATE, RF_Purify_ABSOLUTE, ESTIMATE, ABSOLUTE and LUMP

To compare the RF based models with the methods of which they were derived, we went on to calculate the correlations of RF_Purify_ESTIMATE and RF_Purify_ABSOULTE with the ABSOLUTE, ESTIMATE and LUMP purities which are available for the TCGA dataset (Fig. 2): As expected, the correlations between RF_Purify_ESTIMATE and ESTIMATE as well as RF_Purify_ABSOLUTE and ABSOLUTE were high (0.88 and 0.89 respectively) but also the comparison with the LUMP method yielded a high degree of concordance (correlation coefficient: 0.73/0.74 for RF_Purify_ESTIMATE/RF_Purify_ABSOLUTE). We thus concluded that the two models were able to reliably extrapolate the ESTIMATE and ABSOLUTE tumor purity measures on our test set of TCGA samples.

**Fig. 3:**
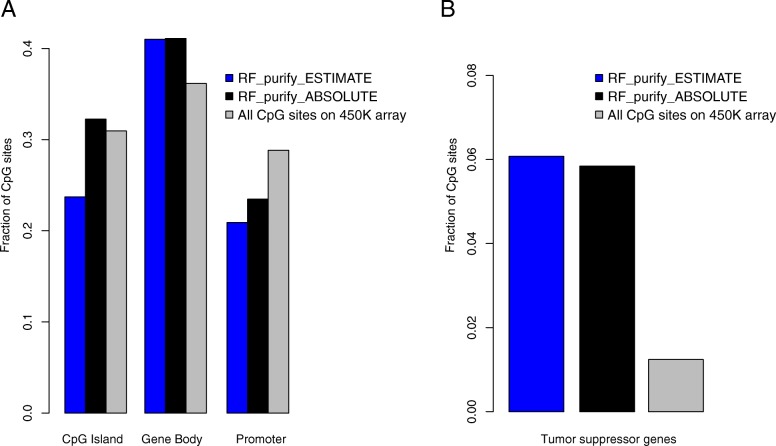
Characterization of RF_purify_ESTIMATE and RF_purify_ABSOLUTE. Figure 3 A displays the fraction of CpG sites localized in CpG islands, gene bodies and promoters in the two models compared to all CpG sites on the 450 K array. Figure 3 B the fraction of CpG sites that overlap with tumor suppressor genes

Seeking to further characterize the CpG sites which are the predictors in both RF models, we analyzed the distribution of CpG sites in the genome compared to all CpG sites on the 450 k array (Fig. 3 A): There was a higher fraction of CpG sites localized to the gene body when compared to all probes on the array (0.41 in both RF models, 0.36 for all CpG sites). More importantly, we find that a higher fraction of CpG sites overlaps with tumor suppressor genes in both RF based models (Fig. 3 B, 0.06 for RF_purify_ESTIMATE and 0.058 for RF_purify_ABSOLUTE and 0.012 for all CpG sites on the array), among these are important transcription factors such as SOX1 and PAX6 in RF_purify_ABSOLUTE as well as RUNX1 and also PAX6 in RF_purify_ESTIMATE, to name a few (a full list is provided as Additional file 6: Table S2). This supports the notion that CpG sites which localize to tumor suppressor genes may be helpful in distinguishing between DNA contributed from neoplastic and non-neoplastic cells in a tumor-stroma admixture.

While correlation between the RF_models and ESTIMATE/ABSOLUTE is a helpful overall measure of quality, the absolute differences in estimated purities may in some instances be more helpful to judge if there is a high overall discrepancy between models. We therefore also compared the absolute differences in tumor purities: The median differences between RF model and the corresponding gold standard were 0.01 for ESTIMATE and 0.009 for ABSOLUTE (Additional file 3: Figure S3).

### Application of the model and orthogonal validation methods to the pediatric brain tumor methylation data

Having fitted our two Random Forest regression models on the TCGA dataset, we next applied the method to the previously published dataset from Capper et al. which contains methylation array data on the most important central nervous system tumors [1]. Although ABSOLUTE purity values based from WES or SNP array data are not available for these samples, a subset of these tumors has been characterized by gene expression arrays and we calculated ESTIMATE scores for these tumors. Thus, we used this dataset as a bona fide orthogonal validation of our RF based methods.

**Fig. 4:**
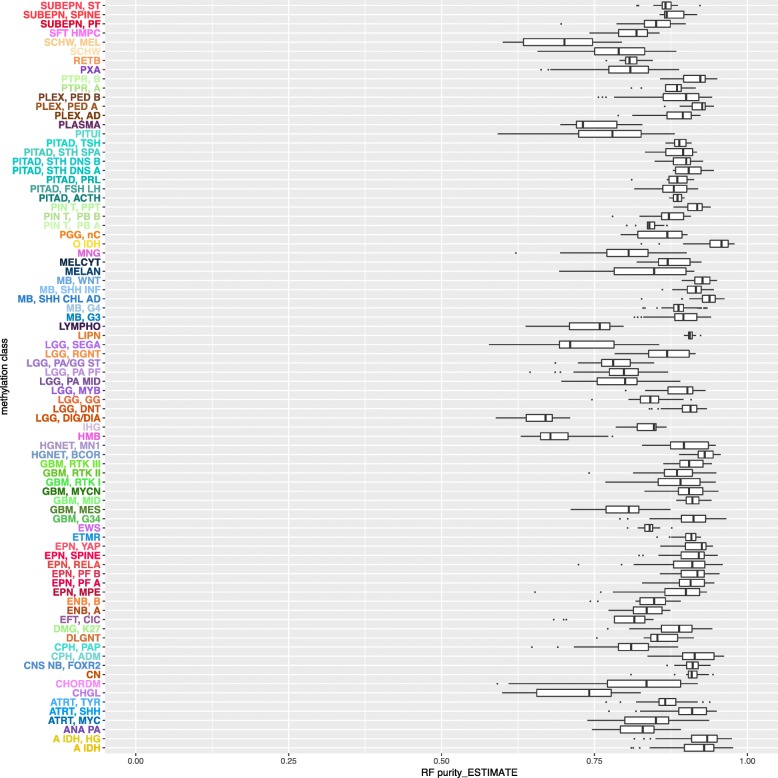
Tumor purities in different entities and their subgroups (Capper et al. [4]) as calculated by RF_Purify_ESTIMATE

Using the RF_Purify approach, we did not only find relevant differences between the various tumor entities but also between subgroups of tumor entities (Fig. 4):

Notably, tumor purity was highest in Medulloblastoma among all entities examined, with the WNT-subgroup displaying the highest RF_Purify_ESTIMATE and RF_Purify_ABSOLUTE scores. Reports on tumor purity in brain tumors specifically are sparse and most studies have rather aimed at delineating spatial, genetic homogeneity in tumor biopsies [12]. Interestingly, Atypical teratoid rhabdoid tumor (ATRT) which is a genetically homogeneous disease but often perceived as histologically heterogeneous had purities rather in the lower spectrum, which correlates with its pleomorphic, histopathological appearance. The ATRT-MYC subgroup – characterized by a higher degree of immune cell infiltration than the two other subgroups ATRT-TYR and ATRT-SHH [2] – was found to display the lowest average purity (mean RF_Purify_ESTIMATE score: 0.837).

Grossly, glial tumors displayed a lower tumor purity than embryonal ones- although in some entities, we discovered relevant subgroup specific differences: GBM-MES exhibited the lowest tumor (mean RF_Purify_ESTIMATE score: 0.801) purity from all glioblastoma samples. This is in line with the upregulation of stromal and immune signatures which is characteristic for these tumors.

To validate the tumor predictions by the RF models, we retrieved the Affymetrix data for a subset of tumors (*n* = 398) and calculated ESTIMATE tumor purity scores. We found both a tight correlation of RF_Purify_ESTIMATE scores and ESTIMATE (R = 0.76) and RF_Purify_ABSOLUTE and ESTIMATE (R = 0. 75).

In this dataset, both the ESTIMATE and the RF_Purify_ESTIMATE method tended to systematically indicate a higher Tumor purity than the RF_Purify_ABSOLUTE.

Overall, the RF_Purify approach allowed for the extrapolation of the ABSOLUTE technique to methylation array datasets not covered in the TCGA projects and has provided insight into tumor purity distributions among a wide range of mostly pediatric brain tumors.

## Discussion

Methylation array based tumor classification is becoming more and more widely used in the clinical setting. The idea to assess tumor purity from methylation data is based on an important observation: The number of probes with intermediate methylation level is greater in tumors compared to normal samples. Many of these sites which display intermediate methylation levels are the result of tumor infiltration by non-neoplastic cells. Thus, tumor (im) purity is an important latent variable which has the potential to confound statistical analysis. While several other methods have been published (InfiniumPurify [13]/ PAMES) the majority of these rely on the use of non-neoplastic tissue as control. This approach however is error prone as in many cases no appropriate control is available and the cell of origin of many tumors is either not known or not available.

We present a Random Forest based approach to estimate tumor purity. Beyond the TCGA data, we have applied tumor purity estimation to the methylation data in Capper et al. (2018) – this is a crucial step given that the vast majority of models which are available are strongly focused on the entities represented in TCGA. Based on the quantile tables presented here (Additional file 5: Table S1), these data allow for the delineation of cut-offs per entity which are able to sort out low-purity samples. More specifically, we have shown that tumor purity not only varies with the entity chosen but in some cases (such as high grade gliomas and medulloblastomas) is also depending on the subgroup of the respective entity.

A critical caveat of the RF_Purify models is the choice of the ABSOLUTE and ESTIMATE method as gold standards. The methods that we present display high concordances with the reference methods and are thus also prone to the same systemic biases which may affect either ABSOLUTE and ESTIMATE. Thus, as a potential concern, RF_Purify may systematically fail to estimate tumor purity in tumor subtypes not covered by the TCGA data set. This concern was not justified - the high correlation of RF_Purify_ESTIMATE and ESTIMATE in the set from Capper et al. indicates that RF_Purify generalizes to other entities not covered by TCGA. As a general observation, RF_Purify_ABSOLUTE scores were lower than RF_Purify_ESTIMATE scores both in the TCGA dataset and the Capper et al. data. This reproduces a systemic difference which can also be observed in the TCGA data.

Thus it is more important to consider the calculated purity of a give sample in relation to other samples from the same tumor (sub-) group (reference values are provided in Additional file 5: Table S1) and not aim at the absolute purity value- in particular given the systematic differences between ABSOLUTE and ESTIMATE (Fig. 1, Additional file 1: Figure S1).

Beyond providing a reference to exclude low purity samples from the analyses, the identification of entities and subgroups of entities with a low tumor purity may hold the promise of identifying entities with a high number of immune cells that infiltrate tumors and ultimately to identify entities which are thus amenable for immunotherapy.

## Conclusions

We have shown that our model can also be applied to non TCGA datasets, yielding tumor purity estimations that correlate well with purities, estimated by different techniques.

Taken together, estimated tumor purity using our model is a potential helpful sample quality measure the can be accounted for by batch adjustment methods or by including it in statistical models, for example in differential expression, proteomic analysis [14], or QTL screening to name a few.

## Methods

We aimed at generating two separate RF models, which are able to extrapolate the gold standard ABSOLUTE [4] and ESTIMATE methods (Additional file 2: Figure S2 shows an overview on the methodology workflow). As a first step, we downloaded the available 450 K methylation array data for all TCGA samples (https://cancergenome.nih.gov/). The raw data was subjected to the same preprocessing steps as highlighted in Capper et al. and beta values were calculated accordingly.

For deriving the training and test set to generate RF_purify_ABSOLUTE we downloaded all available ABSOLUTE values from the TCGA dataset (2308 samples) and for RF_purify_ESTIMATE we used all samples with available ESTIMATE values (6343 samples). We split each of these datasets into a training set (70% of all samples) and a test set (30% of all samples) using the function “createDatapartition” (R-library caret, v 6.0–83). For the RF_Purify_ABSOLUTE, the training set consisted of 1617 samples, for the RF_Purify_ESTIMATE of 4452 samples.

To exclude the possibility that certain entities are underrepresented in the training or test set, we checked the representation of these (Additional file 4: Figure S4) and found a proportional representation of each cancer type.

To reduce the number of predictors used for final Random Forest modelling, we applied Hartigan’s Dip test to each training set independently. This procedure identifies CpG sites which follow a multimodal distribution and is thus thought to better identify probes with intermediate levels of methylation that may stem from increased stromal infiltration in the tumor [15].

In previous studies investigating tumor purity, it was inferred that these CpG sites were most predictive for a non-tumor cell infiltration. We tested different numbers of predictors (top 5, 10, 20, 30% of all CpG sites) for this first step of variable reduction and executed all further steps of model generation using these different numbers of predictors: Consistently, we found that the out of bag error of the subsequently trained models was lowest when using the top 20% of CpG sites selected by Hartigan’s diptest. This also held true when comparing the diptest to choosing the top 5,10,20 and 30% CpG sites with the highest standard deviation.

After this initial step of variable reduction, a two step random forest procedure was applied to both training datasets using the randomForest function (R package: randomForest): The first RF step, performed with *n* = 500 trees, served to further reduce the number of CpG sites. Thereafter the predictors (CpG sites) were ranked according to the built-in importance measure of the RF function.

To optimize this preliminary model, we generated further RF_models with various numbers of CpG sites (0.1, 1, 5 and 10%), calculated the tumor purities of the training sets for each of these models and chose the model which minimized the out-of-bag error. Finally, both for the ESTIMATE and the ABSOLUTE based methods, models with numbers of 856 CpG sites proved to be the model with the lowest number of predictors used but still with a low out-of-bag error.

The second RF step thus finalized both methods. The final versions of the models are available in an R-package at https://github.com/mwsill/RFpurify.

To further characterize the CpG sites which act as predictors in the two models, we used the annotations from the R-package IlluminaHumanMethylation450kanno.ilmn12.hg19 and tested how many CpG sites which were represented in the models overlapped Promoters, Gene bodies and CpG islands. To annotate CpG sites and gene symbols, we also used this database. For quantification of the overlap with tumor suppressor genes (TSG), we downloaded a list of tumor suppressor genes from the database TSG2.0 (web page https://bioinfo.uth.edu/TSGene) and overlapped these TSG with the gene annotations derived from R-package IlluminaHumanMethylation450kanno.ilmn12.hg19.

To orthogonally validate the models in a dataset outside of TCGA, we used the methylation array data from Capper et al. which were available in house and corresponding gene expression data (AffymetrixU133 arrays) in 398 samples. For the gene expression data we calculated ESTIMATE purity scores (R-package ESTIMATE: https://bioinformatics.mdanderson.org/estimate/rpackage.html) and subsequently the mean squared error and pearson correlation coefficients between the RF_Purify_ESTIMATE and RF_Purify_ABSOLUTE purities and the ESTIMATE scores.

## Additional files

Additional file 1: **Figure S1.** Pearson correlation of the ABSOLUTE purity values and RF_Purify_ABSOLUTE for the different TCGA tumor entities (A-J) and for the whole TCGA set with ESTIMATE values available (K). (PDF 127 kb)

Additional file 2: **Figure S2.** Overview on the workflow of model generation used to create the two RF based models. (PDF 685 kb)

Additional file 3: **Figure S3.** Histograms show the absolute differences between the RF_purify estimated tumor purity and the ESTIMATE (a) and ABSOLUTE (b) values of the TCGA dataset. (PDF 31 kb)

Additional file 4: **Figure S4.** Barplots show the representation of each TCGA entity in a) RF_purify_ESTIMATE and b) RF_purify_ABSOLUTE: Y-axis denotes the percentage of samples which belong to a certain entity as compared to the whole set. Blue bars denote the training set, grey bars the whole TCGA set for which either ESTIMATE (A) or ABSOLUTE (B) values were available. (PDF 39 kb)

Additional file 5: **Table S1.** Quantiles for the tumor purities as inferred by RF_Purify_ESTIMATE and RF_Purify_ABSOLUTE. (XLSX 16 kb)

Additional file 6: **Table S2.** Tumor suppressor genes which overlap with the CpG sites of RF_Purify_ESTIMATE and RF_Purify_ABSOLUTE, (XLSX 11 kb)

Additional file 7: **Table S3.** Abbreviations for the entities represented in the TCGA dataset and in Capper et al. (XLSX 13 kb)

## Data Availability

The R-package for this method is available at https://github.com/mwsill/RFpurify

## Abbreviations

DMR: Differentially methylated region
LUMP: Leukocyte unmethylation for purity
QTL: Quantitative trait loci
SNP: Single nucleotide polymorphism
TCGA: The cancer genome atlas
TSG: Tumor suppressor gene
