# Supplementary figures and images for: RF_Purify: a novel tool for comprehensive analysis of tumor-purity in methylation array data based on random forest regression
Source: BMC Bioinformatics. 2019 Aug 16;20:428. doi: 10.1186/s12859-019-3014-z (PMC6697926; doi:10.1186/s12859-019-3014-z)

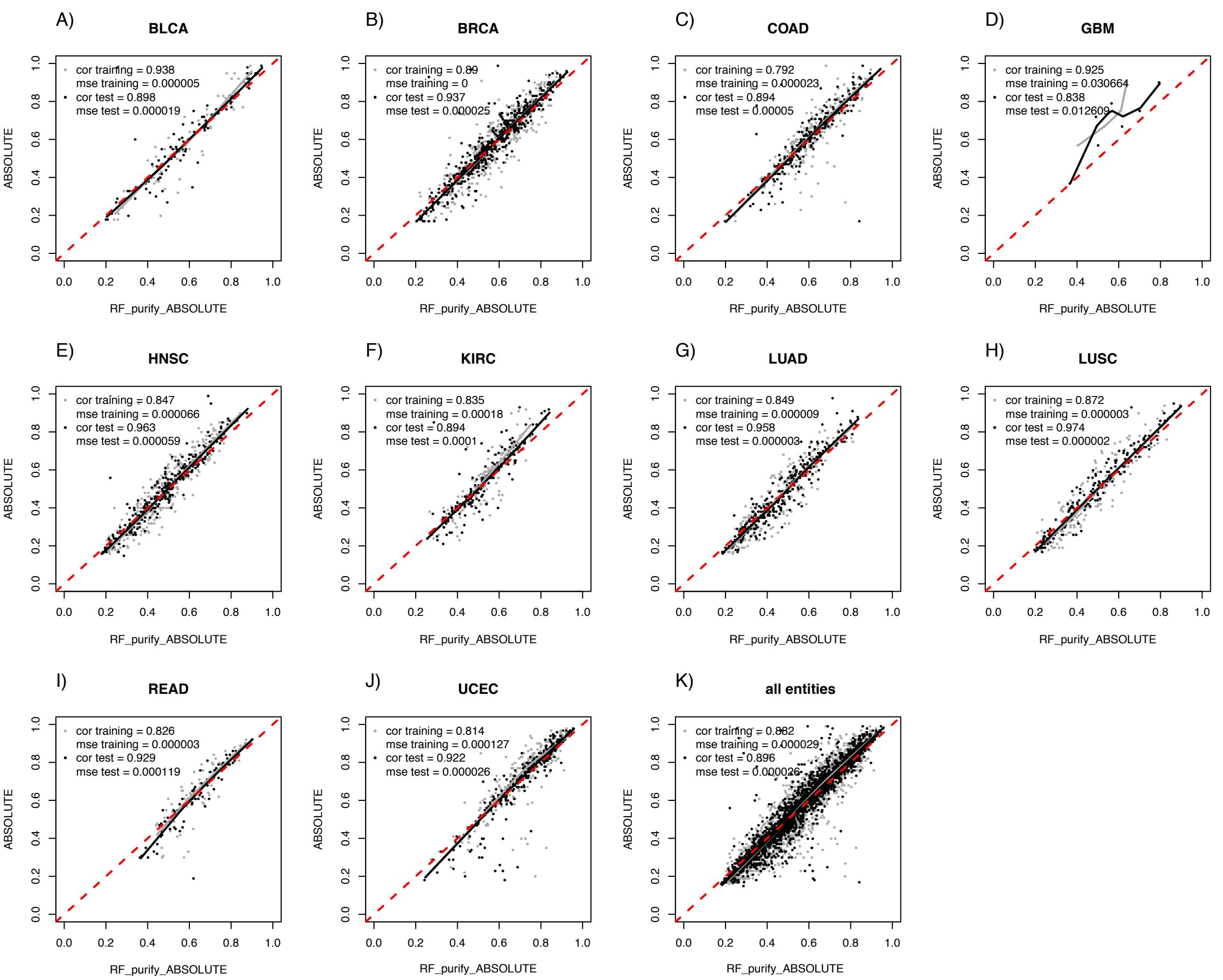

Supplement: Supplementary file 1 — Figure S1. Pearson correlation of the ABSOLUTE purity values and RF_Purify_ABSOLUTE for the different TCGA tumor entities (A-J) and for the whole TCGA set with ESTIMATE values available (K). (PDF 127 kb) [file 12859_2019_3014_MOESM1_ESM.pdf]

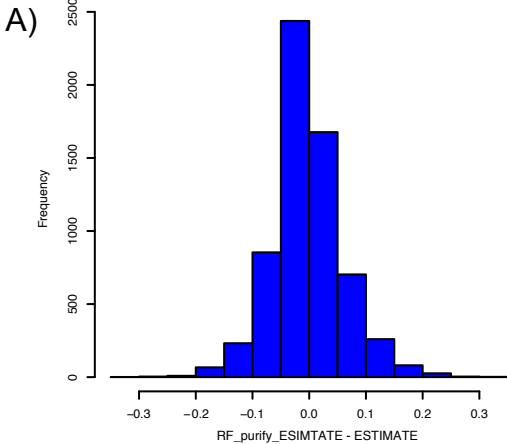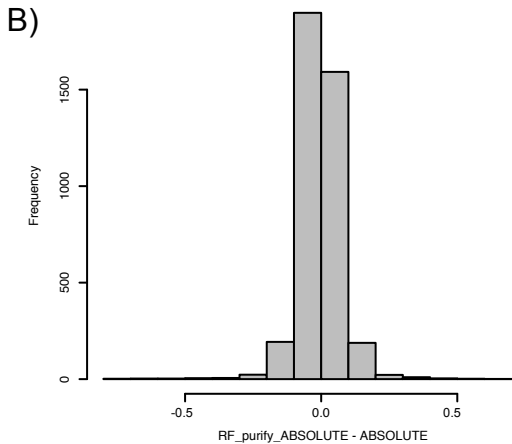

Supplement: Supplementary file 3 — Figure S3. Histograms show the absolute differences between the RF_purify estimated tumor purity and the ESTIMATE (a) and ABSOLUTE (b) values of the TCGA dataset. (PDF 31 kb) [file 12859_2019_3014_MOESM3_ESM.pdf]

A)

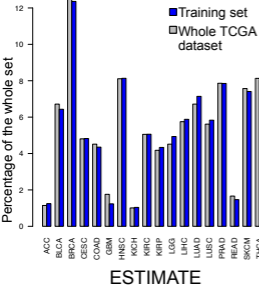

B)

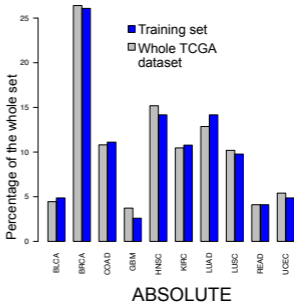

Supplement: Supplementary file 4 — Figure S4. Barplots show the representation of each TCGA entity in a) RF_purify_ESTIMATE and b) RF_purify_ABSOLUTE: Y-axis denotes the percentage of samples which belong to a certain entity as compared to the whole set. Blue bars denote the training set, grey bars the whole TCGA set for which either ESTIMATE (A) or ABSOLUTE (B) values were available. (PDF 39 kb) [file 12859_2019_3014_MOESM4_ESM.pdf]
