# Supplementary material for: RF_Purify: a novel tool for comprehensive analysis of tumor-purity in methylation array data based on random forest regression
Source: BMC Bioinformatics. 2019 Aug 16;20:428. doi: 10.1186/s12859-019-3014-z (PMC6697926; doi:10.1186/s12859-019-3014-z)
Supplement: Supplementary file 2 — Figure S2. Overview on the workflow of model generation used to create the two RF based models. (PDF 685 kb) [file 12859_2019_3014_MOESM2_ESM.pdf]

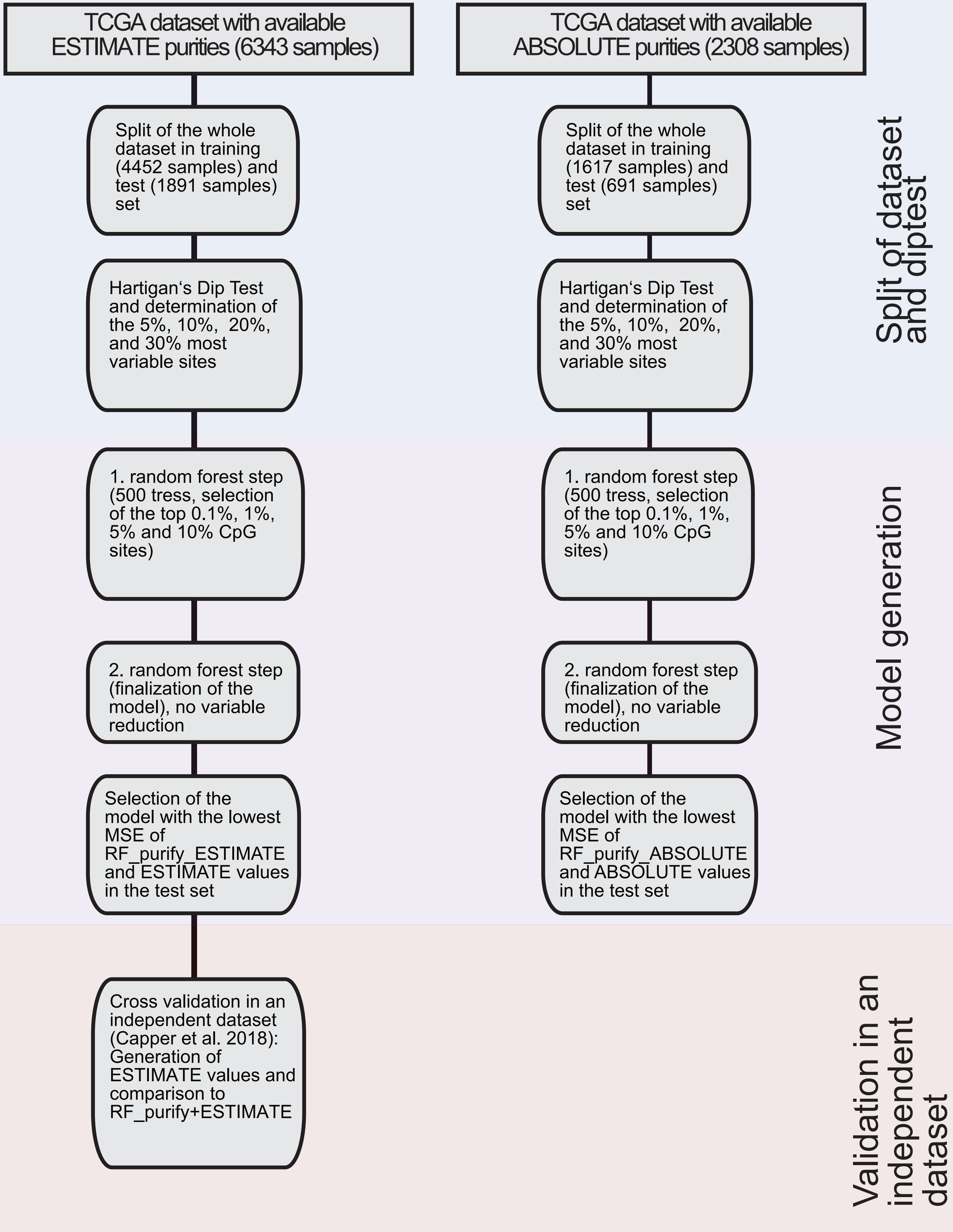

TCGA dataset with available ESTIMATE purities (6343 samples)
TCGA dataset with available ABSOLUTE purities (2308 samples)
Split of the whole dataset in training (4452 samples) and test (1891 samples) set
Split of the whole dataset in training (1617 samples) and test (691 samples) set
Hartigan's Dip Test and determination of the 5%, 10%, 20%, and 30% most variable sites
Hartigan's Dip Test and determination of the 5%, 10%, 20%, and 30% most variable sites
Split of dataset and diptest
1. random forest step (500 tress, selection of the top 0.1%, 1%, 5% and 10% CpG sites)
1. random forest step (500 tress, selection of the top 0.1%, 1%, 5% and 10% CpG sites)
2. random forest step (finalization of the model), no variable reduction
2. random forest step (finalization of the model), no variable reduction
Selection of the model with the lowest MSE of RF_purify_ESTIMATE and ESTIMATE values in the test set
Selection of the model with the lowest MSE of RF_purify_ABSOLUTE and ABSOLUTE values in the test set
Model generation
Cross validation in an independent dataset (Capper et al. 2018): Generation of ESTIMATE values and comparison to RF_purify+ESTIMATE
Validation in an independent dataset
